# ShiftCrypt: a web server to understand and biophysically align proteins through their NMR chemical shift values

**DOI:** 10.1093/nar/gkaa391

**Published:** 2020-05-27

**Authors:** Gabriele Orlando, Daniele Raimondi, Luciano Porto Kagami, Wim F Vranken

**Affiliations:** Interuniversity Institute of Bioinformatics in Brussels, ULB-VUB, Triomflaan, Brussels 1050, Belgium; Switch Laboratory, VIB, Leuven, Belgium; ESAT-STADIUS, KU Leuven, Kasteelpark Arenberg 10, 3001 Leuven, Belgium; Interuniversity Institute of Bioinformatics in Brussels, ULB-VUB, Triomflaan, Brussels 1050, Belgium; Interuniversity Institute of Bioinformatics in Brussels, ULB-VUB, Triomflaan, Brussels 1050, Belgium; Structural Biology Brussels, Vrije Universiteit Brussel, Pleinlaan 2, Brussels 1050, Belgium; VIB Structural Biology Research Centre, Pleinlaan 2, Brussels 1050, Belgium

## Abstract

Nuclear magnetic resonance (NMR) spectroscopy data provides valuable information on the behaviour of proteins in solution. The primary data to determine when studying proteins are the per-atom NMR chemical shifts, which reflect the local environment of atoms and provide insights into amino acid residue dynamics and conformation. Within an amino acid residue, chemical shifts present multi-dimensional and complexly cross-correlated information, making them difficult to analyse. The ShiftCrypt method, based on neural network auto-encoder architecture, compresses the per-amino acid chemical shift information in a single, interpretable, amino acid-type independent value that reflects the biophysical state of a residue. We here present the ShiftCrypt web server, which makes the method readily available. The server accepts chemical shifts input files in the NMR Exchange Format (NEF) or NMR-STAR format, executes ShiftCrypt and visualises the results, which are also accessible via an API. It also enables the ”biophysically-based” pairwise alignment of two proteins based on their ShiftCrypt values. This approach uses Dynamic Time Warping and can optionally include their amino acid code information, and has applications in, for example, the alignment of disordered regions. The server uses a token-based system to ensure the anonymity of the users and results. The web server is available at www.bio2byte.be/shiftcrypt.

## INTRODUCTION

Nuclear magnetic resonance (NMR) spectroscopy provides valuable information on the behaviour of proteins in solution, especially when these proteins dynamically move between different conformations (1). The atom-connected chemical shift values are of particular importance, as they are the most commonly determined and also publicly available from the BioMagResBank (2). They are a measure of the effect of local environment of atoms on their resonance frequency, and contain a mixture of different types of information in relation to the conformation and dynamics of the amino acid residue they are part of (3). Chemical shifts have already been used to estimate protein backbone dynamics (4), secondary structure propensities (5) and torsion angles (6), and chemical shifts derived predictors have been used in several bioinformatics tools (7–9). However, directly relating chemical shifts to structural or conformation-related features might be reductive: chemical shifts contain an heterogeneous mixture of information because they derive from the transient interaction between atoms in space, and linking them to single observable characteristics necessarily leads to a loss of part of the information they contain. Moreover, each amino acid residue contains multiple atoms, each one linked to a chemical shift value, making the data intrinsically multidimensional. Such intra-residue chemical shifts are also highly cross-correlated with each other, and therefore not trivial to handle in terms of data analysis. We recently developed ShiftCrypt, an unsupervised neural network (NN) used to transform chemical shift data (10). ShiftCrypt is based on an auto-encoder architecture and compresses the chemical shift information of the atoms that compose a residue in a single, interpretable, amino-acid type independent value. This compressed value correlates with several structural characteristics, and changes in accordance with changes in the protein’s state, for example in residues involved in dimerisation interactions. Since this tool can be used in order to extract the chemical shift information associated to every residue of a protein, it can be used to interpret the behaviour of proteins in their native soluble state, finding similarities and differences in diverse proteins or within the same protein in different experimental or biological conditions.

We here present the ShiftCrypt web server, which makes the tool available for non-expert users. It parses input files containing chemical shifts in the NMR Exchange Format (NEF) as well as archive NMR-STAR files from the BioMagResBank, executes ShiftCrypt and visualises the results, which are also accessible via an Application Programming Interface (API). Since the ShiftCrypt values reflect the in-solution biophysical state of amino acids in proteins (10), we included a novel option that enables a “biophysically-based” pairwise alignment of two proteins based on their ShiftCrypt values. This approach uses a Dynamic Time Warping approach and can optionally include their amino acid code information. and has applications in, for example, the alignment of disordered regions. The web server is available at http://bio2byte.be/shiftcrypt and uses a token-based system to ensure the anonymity of users and results.

## MATERIALS AND METHODS

### Learning dataset

The dataset that has been used to fit the model consists of 3385 filtered BMRB entries containing NMR chemical shifts. The chemical shifts have been re-referenced using the VASCO approach (11). In order to reduce the effect of mis-assigned or mis-referenced atoms, we removed all the atoms with chemical shift values greater than or lower than respectively the 99th and 1st percentile. For this paper we created three additional datasets: the first one consists of 23 proteins for which the chemical shifts were determined in presence of urea, which partially of completely denatures them. The second one is a collection of 2976 proteins for which the chemical shifts have been calculated in their native state. The last one is composed of 40 proteins randomly selected from the aforementioned native state dataset. All datasets are available from the web server.

### Data encoding and preprocessing

To compress the chemical shift information in a single value, we first need to define which atoms should be taken into consideration for each residue type, so defining an encoding scheme. ShiftCrypt requires a large amount of data in order to be effectively trained, and we therefore discard atoms for which few chemical shifts are available for that amino acid residue type. In our web server we provide three encoding schemes, all of which have been described previously (10):

- Full Atom Model: A model which uses the full list of atoms for which chemical shifts are usually available (see Supplementary Table S1 for the complete list of atoms taken into consideration).
- Reduced Atom Model: A model that only takes H, HA, CA, N, CB and C atoms into consideration. This model is useful to deal with proteins for which less chemical shift information is available. This is also considered the default model in the web server.
- Dimers Model: This model only uses the chemical shifts of CA, N and H atoms and has been shown to be sensitive for the detection of the oligomeric state of proteins (10)

The selection of the model to use for the ShiftCrypt calculation can be performed at the 'data input' page using a drop-down menu.

### Auto-encoder transformation

ShiftCrypt compresses the chemical shift information in a single value using a neural network with auto-encoding architecture (12). The network is structured in two modules: an encoder and a decoder. The first one takes the chemical shifts of each residue as input and passes them to a single neuron with sigmoid activation, the second one takes the value of this neuron as input and tries to replicate the original data. The neural network is then trained to minimise the difference between the original chemical shifts and the encoded-decoded values. The aforementioned neuron with sigmoid activation acts as bottleneck and will provide a mapping of the chemical shift data in a single dimension that minimises the loss of information and provides the ShiftCrypt values. Analysis of the stability of the model and the link between compressed chemical shift values and biophysical characteristic of protein behaviour can be found in the original paper (10)

### Chemical shift based alignment

Biophysical properties have already been used as additional information to increase the quality of protein alignments (13). Chemical shifts can be determined for any soluble protein, even disordered or unstructured ones, with protein size the main experimental limitation. We previously showed that ShiftCrypt is capable of capturing regions of homologous proteins that have conformational similarities, even when the sequence identity between those regions is low (10). Since ShiftCrypt only requires chemical shifts, it can deal with both globular and non-globular proteins, opening up the possibility of aligning proteins by their chemical shift-encoded biophysics. To enable this, we implemented a Dynamic Time Warping-based alignment algorithm that is available in the server. The algorithm minimises the mean euclidean distance between the two ShiftCrypt profiles using a fixed gap penalty (set to 0.4). It is implemented in Python, and allows the (optional) inclusion of amino acidic information using a rescaled BLOSUM62 substitution matrix, obtained applying the QuantileTransformer provided by the scikit-learn python library to the original BLOSUM62 scores and inverting them. The algorithm allows a direct comparison of the in-solution behaviour of proteins and, in principle, can align disordered protein regions by their biophysical characteristics. The alignment score it provides defines the quality of the alignment and must be interpreted as a ‘cost’, meaning that lower values are better, higher ones worse.

### Website and API

The ShiftCrypt website is implemented in the Python Django framework, including a RESTful web service. Protein chemical shift data have to be uploaded one at a time as NMR Exchange format (NEF) (14) or as archive NMR-STAR files from the BioMagResBank. Via a token-based system the user can track their (multiple) uploaded entries fully anonymously without entering their email address. Via an alignment page two entries can be selected to be aligned by their ShiftCrypt values, or via a combined ShiftCrypt/sequence method. The results are available in interactive pages for single or aligned entries. The RESTful web service enables interaction with these features via a consistent set of HTTP method semantics in conjunction with exclusive URIs (Uniform Resource Identifiers). Each object (set of sequence and ShiftCrypt values) can be obtained by a unique URI (e.g. ’shiftcrypt/ api/[unique-id]’). The data is returned using JavaScript Object Notation (JSON) as the structured data exchange format. Full detailed documentation on how to use the server is available on the web site.

## RESULTS

### ShiftCrypt values for folded and unfolded proteins

**Figure 1.**
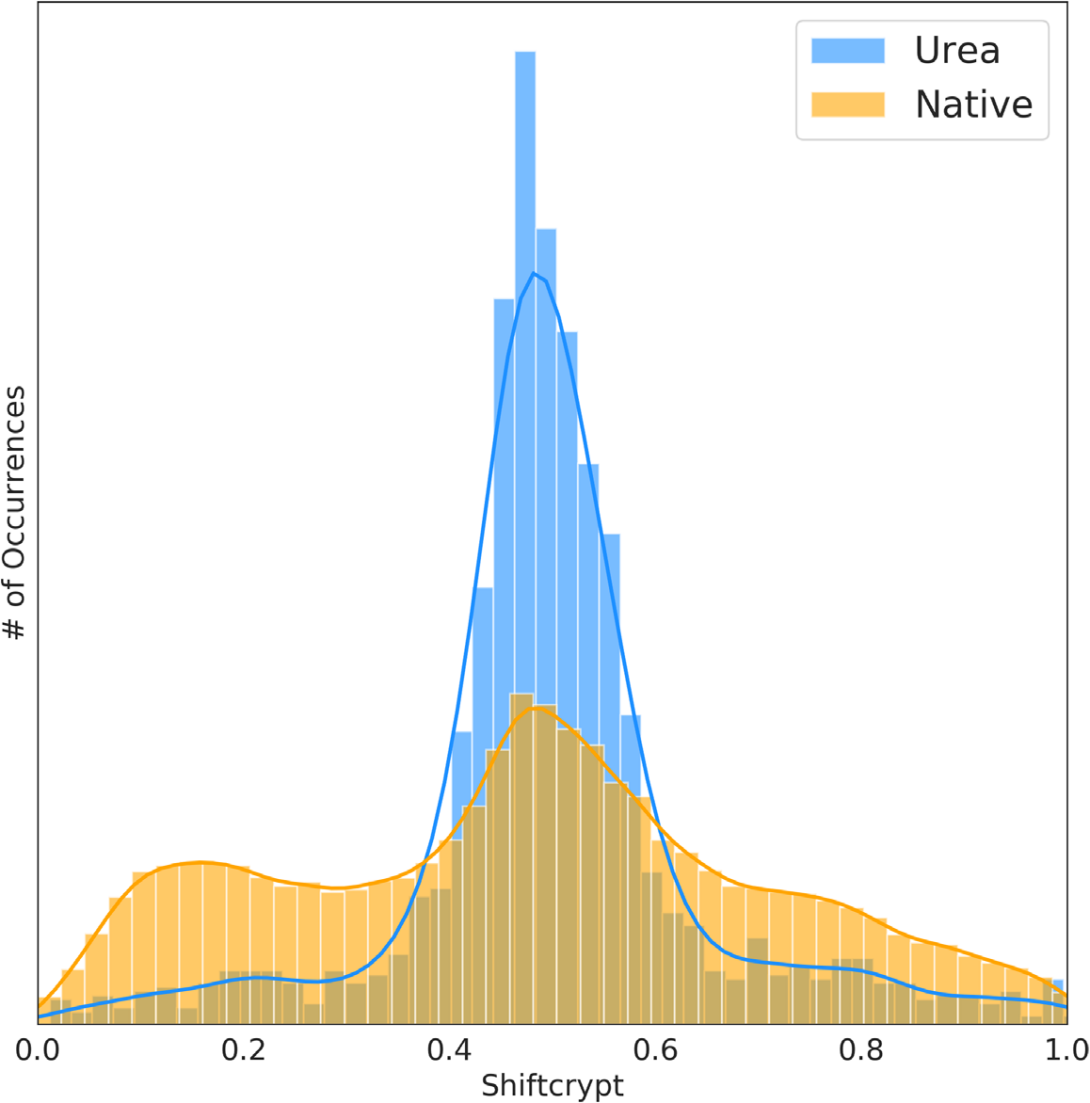
Distribution of the ShiftCrypt values for proteins in urea (blue histogram) and in their native conformation (orange distribution).

We previously demonstrated the link of the single per-residue ShiftCrypt value (10) with protein conformation (4,6) and their evolution over time (dynamics) (15). The distribution of ShiftCrypt values should therefore be very different for folded and unfolded proteins. We compared the ShiftCrypt values of 23 proteins in presence of urea at a range of concentrations, which are expected to be partially or fully unfolded due to urea interference with intramolecular hydrogen bonding, and 2976 proteins in their native conformation, both at similar experimental conditions of pH and temperature (Figure 1).

The two distribution are very different with a p-value between them of 10^−92^ according to the Kolmogorov-Smirnov test. Values around 0.5 are correlated to coil conformation, while low and high values relate respectively to alpha helices and beta sheets (10). As expected, the chemical shifts of proteins in urea are more likely to be found in coil-like conformations. However, there are some residues with high and low shiftCrypt values, indicating they can retain conformational preferences. Since the effect of urea depends on the urea concentration and the nature of the protein, this can in some cases lead to only partial unfolding.

### Residual structures after urea denaturation

**Figure 2.**
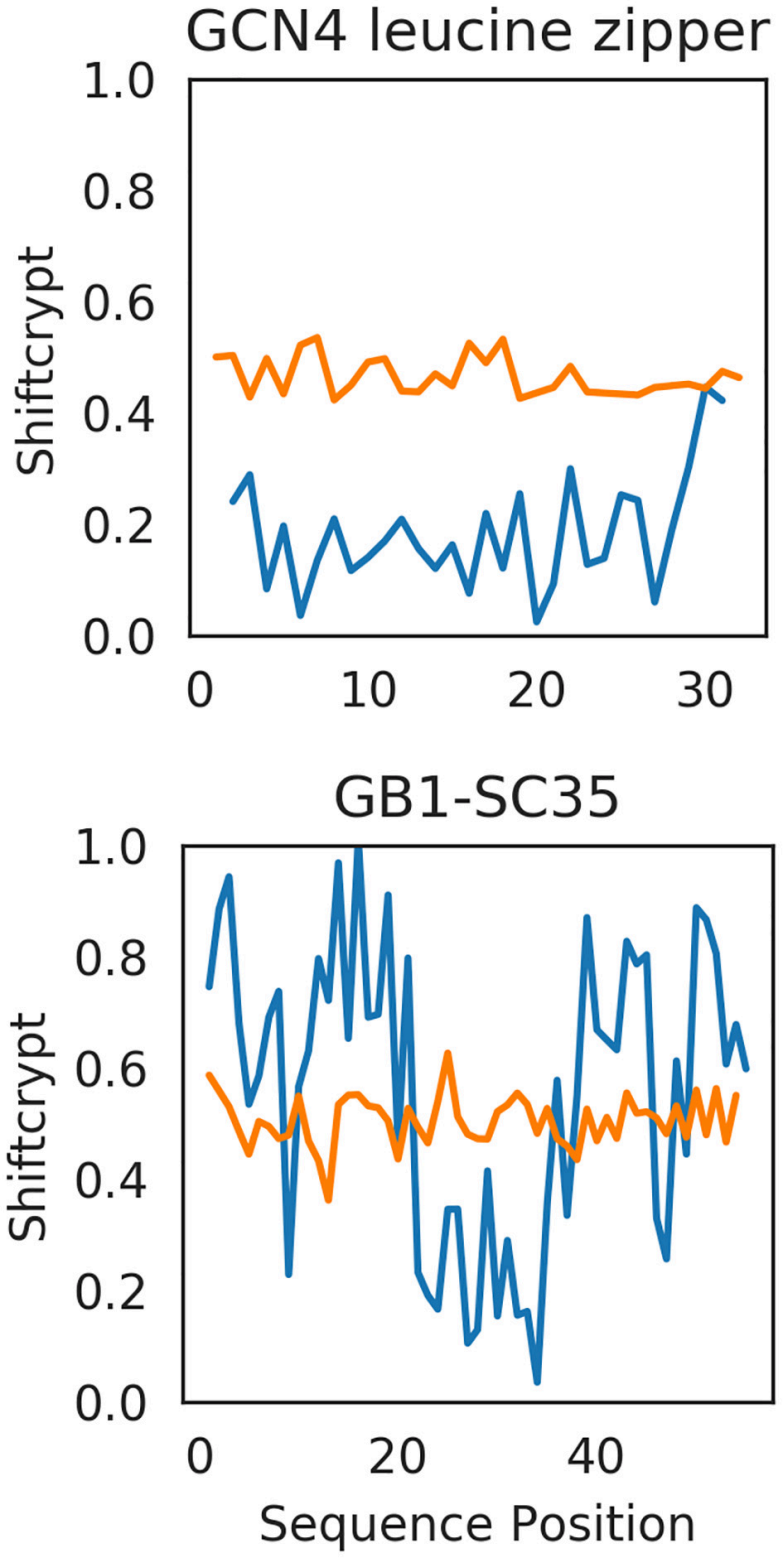
Comparison of the ShiftCrypt profile for two proteins, GB1-SC35 and a GCN4 leucine zipper, in presence (orange) and absence (blue) of urea.

To further illustrate the change from folded to unfolded conformation, we here show two ShiftCrypt case studies of proteins, GB1-SC35 and a GCN4 leucine zipper (a mainly alpha helix artificial protein), using the chemical shifts of the native conformation and the ones with addition of urea (Figure 2), with the aim of visualising the changes in conformation due to urea denaturation.

In both GB1-SC35 and GCN4 leucine zipper the ShiftCrypt profile changes completely in presence of urea, immediately illustrating that these proteins indeed completely unfold with the addition of urea, maintaining almost no local native conformations. There is still a fine-grained profile at the residue level, however, indicating the possibility of distinguishing between the behaviour of residues even when highly disordered.

### Aligning proteins based on their ShiftCrypt values

Aligning proteins without a well defined three dimensional structure, like disordered or partially disordered proteins (16,17), is indeed an open problem. Their sequences are less conserved (18), creating challenges for traditional alignment algorithms. Moreover, they are very dynamic, so that reference structure-based alignments cannot be generated. Chemical shifts, however, contain information about dynamics and conformation, and can indicate transient structural conformations even for unstructured proteins (5). Our web server now allows the global alignment of two proteins based on their ShiftCrypt values, providing both the sequence alignment and an alignment score as output. In order to test the capability of the alignment algorithm to detect similarities between in-solution protein characteristics, we collected the distribution of the alignment scores for three sets of alignments:

- Denatured proteins against denatured proteins: we aligned every protein for which the chemical shifts were extracted in presence of urea against each other.
- Native proteins against denatured proteins: we aligned every protein for which the chemical shifts were extracted in presence of urea against 40 proteins in their native state
- Native proteins against native proteins: we aligned each protein of the aforementioned 40 proteins against each other.

**Figure 3.**
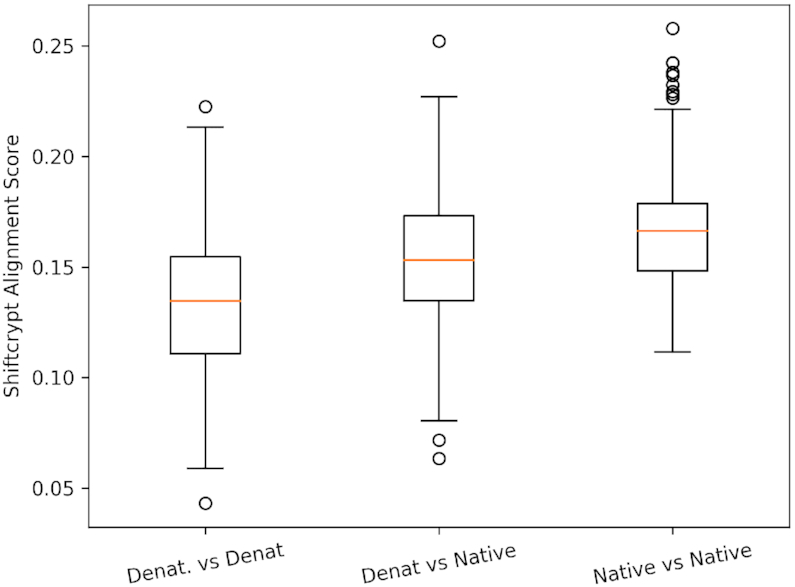
Distribution of the alignment scores based on ShiftCrypt values, with Denat. indicating denatured proteins (by addition of urea), and native proteins in their native conformation. The box-plots represents the distribution of the alignment scores obtained by aligning all sequences between these datasets. The higher the alignment scores, the more diverse the ShiftCrypt profiless.

The distribution of the scores for the three sets of alignments performed (Figure 3) shows that, as expected, the scores are in average lower (better) for the denatured against denatured set, since proteins denatured with urea are mainly unstructured and homogeneous from the conformational point of view. The native-against-native scores are the highest (worst), as here stable secondary structure elements will be very differently distributed. Supplementary Figure S1 shows the distribution of the alignment scores using a sequence based Needleman–Wunsch algorithm. It shows that the difference between the distribution of the scores cannot be explained by the protein sequence similarity.

### An example of chemical shifts-based alignment

**Figure 4.**
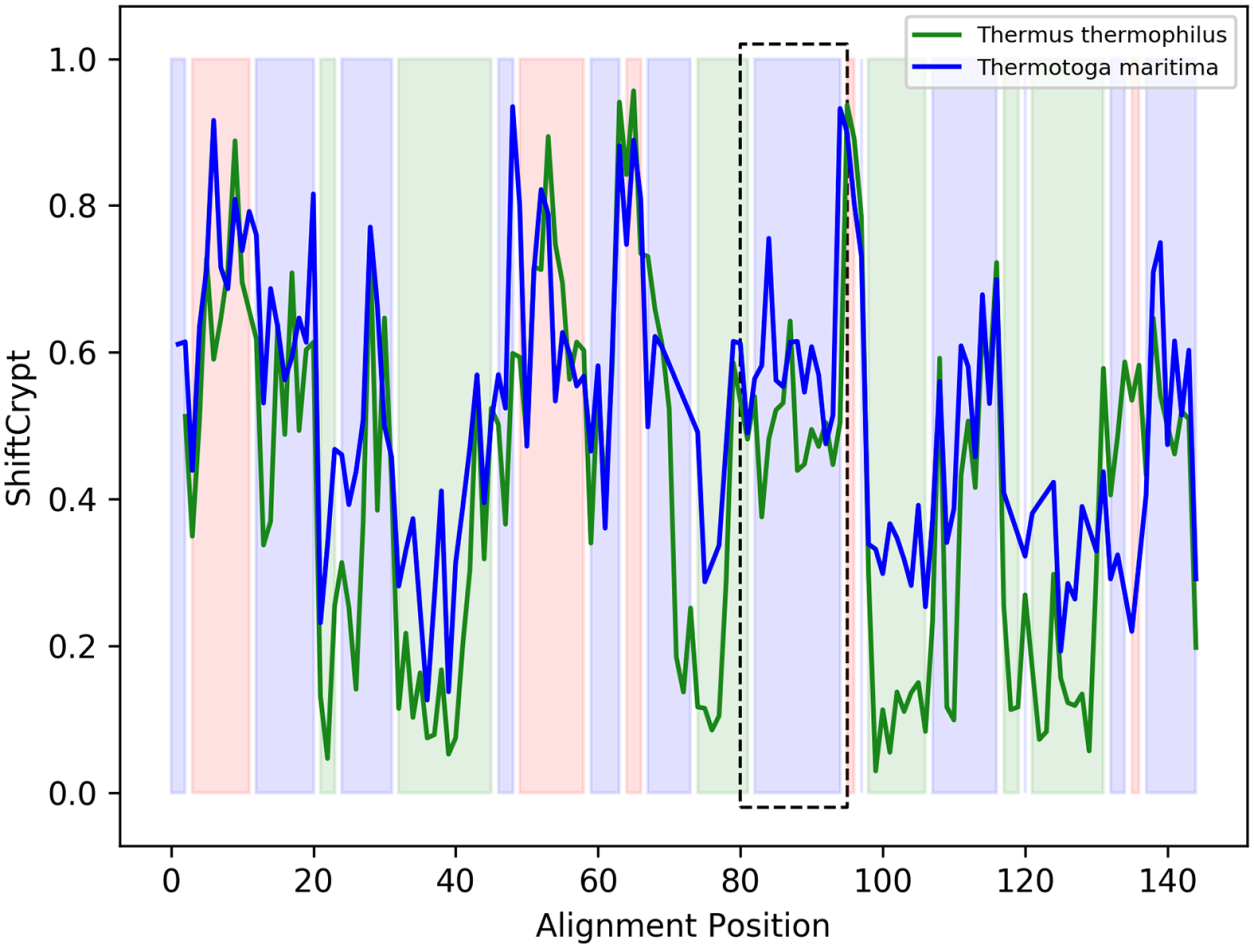
Alignment of the *Thermus thermophilus* (green) and *Thermotoga maritima* (blue) L11 ribosomal proteins obtained using the ShiftCrypt alignment. The coloured regions represent the secondary structure elements of the *T. thermophilus* protein: red for beta sheet, green for alpha helix and blue for coil. The region highlighted with the black box represents a region annotated as intrinsically disordered in the homologous protein of *Geobacillus stearothermophilus*

As mentioned, aligning disordered regions is challenging since sequence conservation is often low and we cannot rely on structure superimposition. As a case study, we here align by ShiftCrypt value two 50S ribosomal L11 proteins, a protein that is part of the ribosome (19), one from *Thermus thermophilus* and the other from *Thermotoga maritima* (BMRB IDs 4965 and 5513 respectively) (Figure 4). Their shared sequence identity is 39%.

These two proteins likely contain an intrinsically disordered region, with one of their homologs, the L11 ribosomal proteins of *Geobacillus stearothermophilus*, having a disorder annotation in the Disprot database (20). This disordered region is highlighted by the black box in the figure, and is correctly aligned. In addition, the alignment shows a more pronounced helical content in *T. thermophilus*, although the final helical region is shorter. Also note the fine-structure within the secondary structure elements, indicating variations of their internal per-residue behaviour. The sequence alignment of the proteins using the pure ShiftCrypt alignment, the ShiftCrypt alignment with the addition of amino acidic information and the pure sequence based alignment is reported in Supplementary file *alignment.fasta*.

## CONCLUSION

The ShiftCrypt web server enables users to compress and interpret chemical shift data with minimal loss of information. The server provides interactive visualisation of the ShiftCrypt results as well as introducing a novel chemical shift-based alignment approach. We show how ShiftCrypt can be used to visualise and detect conformational changes in proteins, here for denaturation in urea, but equally for less drastic changes, as illustrated in the ShiftCrypt method paper. The alignment tool we propose does not rely on sequence or structural similarity between the two target proteins, and allows the detection of similarities and differences between the biophysical behavior of residues in proteins. The web server has a token system that allows storage and the re-utilisation of previously analysed proteins.

## Supplementary Material

gkaa391_Supplemental_Files
