# Supplementary material for: ShiftCrypt: a web server to understand and biophysically align proteins through their NMR chemical shift values
Source: Nucleic Acids Res. 2020 May 27;48(W1):W36–40. doi: 10.1093/nar/gkaa391 (PMC7319548; doi:10.1093/nar/gkaa391)
Supplement: gkaa391_Supplemental_Files [file gkaa391_supplemental_files.zip › supplementary.pdf]

Supplementary Information

ShiftCrypt: a web server to understand and biophysically aligning proteins through their NMR chemical shift values

Gabriele Orlando, Daniele Raimondi, Luciano Kagami, Wim Vranken

Supplementary Table 1: **Encoding scheme of ShiftCrypt for the full atoms model**. The atoms included in the encoding scheme of the full atoms model, per amino acid type. Atoms with redundant values are taken just once.

Residue type	Encoding scheme
A	HA, CA, CB, C, HB2, H, N
C	HA, CA, CB, C, HB2, H, N
E	HA, CA, CB, C, HB2, H, N
D	HA, CA, CB, C, HB2, N, H
G	HA3, HA2, CA, C, N, H
F	HA, CA, CB, C, HB2, H, N
I	HA, CA, CB, C, HB, N, H
H	HA, CA, CB, C, N, H, HB2
K	HA, CA, CB, C, HB2, H, N
M	HA, CA, CB, C, HB2, H, N
L	HA, CA, CB, C, HB2, H, N
N	HA, CA, CB, C, HB2, H, N
Q	HA, CA, CB, C, HB2, H, N
P	HA, CA, CB, C, HG2, HB2, CD, HD2
S	HA, CA, CB, C, HB2, H, N
R	HA, CA, CB, C, HB2, H, N
T	HA, CA, CB, C, HB, N, H
W	HA, CA, CB, C, HB2, H, N
V	HA, CA, CB, C, N, H
Y	HA, CA, CB, C, HB2, H, N

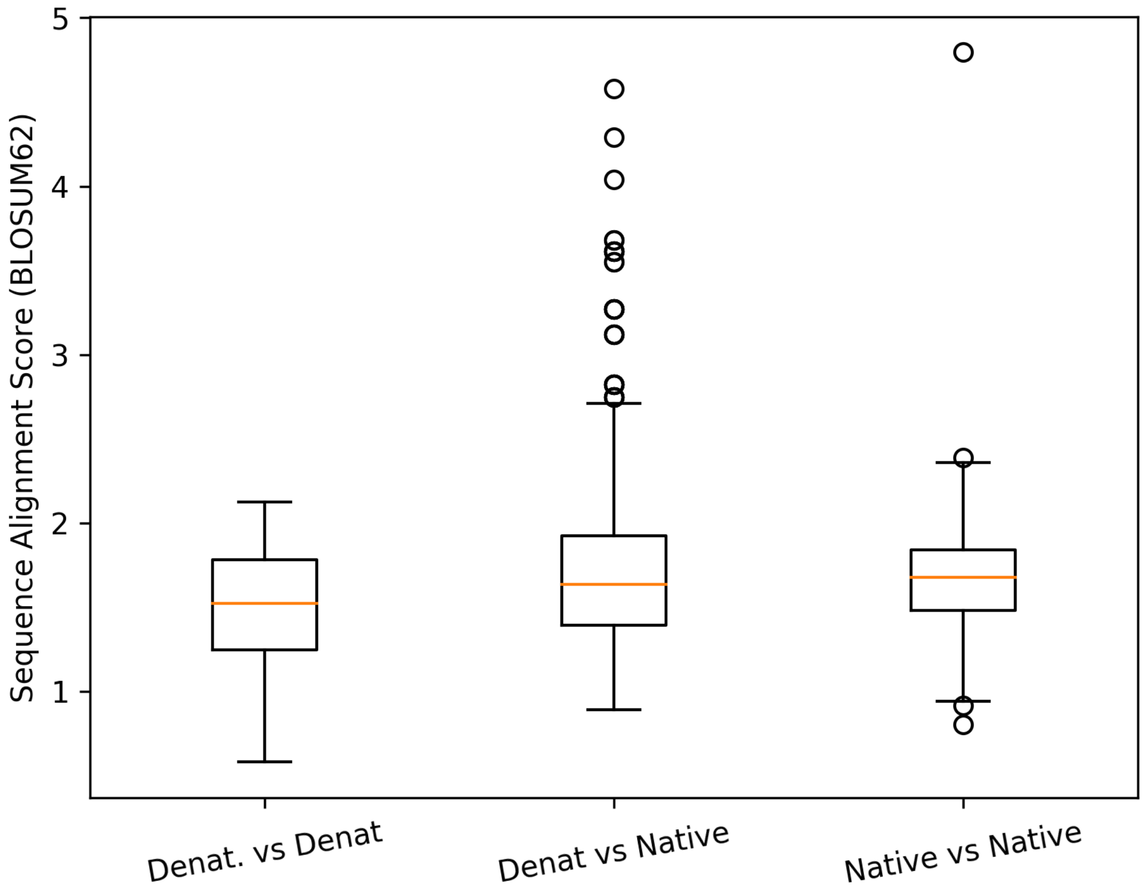

Supplementary Figure 1: A replication of the analysis showed in Figure 3 using sequence based pairwise Needleman-Wunsch alignment to score the quality of the alignment. The alignments have been performed using the BLOSUM62 substitution matrix. The scores are normalized by the alignment length. The higher the score, the better the alignment.
